# *De Novo* Generated Human Red Blood Cells in Humanized Mice Support *Plasmodium falciparum* Infection

**DOI:** 10.1371/journal.pone.0129825

**Published:** 2015-06-22

**Authors:** Anburaj Amaladoss, Qingfeng Chen, Min Liu, Sara K. Dummler, Ming Dao, Subra Suresh, Jianzhu Chen, Peter R. Preiser

**Affiliations:** 1 Infectious Diseases Interdisciplinary Research Group, Singapore-Massachusetts Institute of Technology Alliance for Research and Technology, Singapore, 138602, Singapore; 2 Humanised Mouse Unit, Institute of Molecular and Cell Biology, Agency for Science, Technology and Research, Singapore, 138673, Singapore; 3 Department of Materials Science and Engineering, Massachusetts Institute of Technology, Cambridge, MA, 02139, United States of America; 4 Department of Biomedical Engineering, Carnegie Mellon University, Pittsburgh, PA, 15213, United States of America; 5 Department of Materials Science and Engineering, Carnegie Mellon University, Pittsburgh, PA, 15213, United States of America; 6 The Koch Institute for Integrative Cancer Research and Department of Biology, Massachusetts Institute of Technology, Cambridge, MA, 02139, United States of America; 7 School of Biological Sciences, Nanyang Technological University, Singapore, 637551, Singapore; Instituto de Ciências Biomédicas / Universidade de São Paulo—USP, BRAZIL

## Abstract

Immunodeficient mouse–human chimeras provide a powerful approach to study host specific pathogens like *Plasmodium (P*.*) falciparum* that causes human malaria. Existing mouse models of *P*. *falciparum* infection require repeated injections of human red blood cells (RBCs). In addition, clodronate lipsomes and anti-neutrophil antibodies are injected to suppress the clearance of human RBCs by the residual immune system of the immunodeficient mice. Engraftment of NOD-scid Il2rg^-/-^ mice with human hematopoietic stem cells leads to reconstitution of human immune cells. Although human B cell reconstitution is robust and T cell reconstitution is reasonable in the recipient mice, human RBC reconstitution is generally poor or undetectable. The poor reconstitution is mainly the result of a deficiency of appropriate human cytokines that are necessary for the development and maintenance of these cell lineages. Delivery of plasmid DNA encoding human erythropoietin and interleukin-3 into humanized mice by hydrodynamic tail-vein injection resulted in significantly enhanced reconstitution of erythrocytes. With this improved humanized mouse, here we show that *P*. *falciparum* infects *de novo* generated human RBCs, develops into schizonts and causes successive reinvasion. We also show that different parasite strains exhibit variation in their ability to infect these humanized mice. Parasites could be detected by nested PCR in the blood samples of humanized mice infected with *P*. *falciparum* K1 and HB3 strains for 3 cycles, whereas in other strains such as 3D7, DD2, 7G8, FCR3 and W2mef parasites could only be detected for 1 cycle. *In vivo* adaptation of K1 strain further improves the infection efficiency and parasites can be detected by microscopy for 3 cycles. The parasitemia ranges between 0.13 and 0.25% at the first cycle of infection, falls between 0.08 and 0.15% at the second cycle, and drops to barely detectable levels at the third cycle of infection. Compared to existing mouse models, our model generates human RBCs *de novo* and does not require the treatment of mice with immunomodulators.

## Introduction

Malaria is caused by parasites of the *Plasmodium* species which are transmitted by infected Anopheles mosquitoes. *Plasmodium* species are host specific, making it difficult to model human parasite infection in laboratory animals. Therefore, most *in vivo* experimental studies have been carried out with mouse and rodent *Plasmodium* strains. However, differences in invasion and disease pathology between parasite species remain a major problem. The lack of appropriate experimental models have hampered the evaluation of new drugs and vaccines prior to clinical trials [1].

One approach to overcome this challenge is to supplement severe combined immunodeficient (scid) mice with human RBCs. The resulting mice support a limited blood stage *P*. *falciparum* infection [2–6]. The requirements to inject large volumes of human RBCs repeatedly along with treating mice with anti-neutrophil antibody and clodronate liposomes to suppress the rapid clearance of the injected human RBCs by macrophages makes this a difficult system to work with [7]. NOD-scid Il2rg^-/-^ or NSG mice developed recently bears a targeted mutation at the interleukin-2 receptor (IL-2R) gamma chain locus. Macrophages are functionally immature in NSG mice due to IL2-R gamma chain deficiency [8]. *P*. *falciparum* infection was also reported in NSG mice without the use of clodronate liposomes or anti-neutrophil antibody, but still requiring daily human RBC injection [9]. The best small animal model for malaria infection so far is the human RBC-supplemented, immune cell-optimized humanized (or RICH) mice that support multiple cycles of *P*. *falciparum* infection in the presence of a human immune system [10]. Nevertheless, it is still a formidable challenge to establish a malaria infection model that does not require regular human RBC supplementation.

NSG mice have been reported to support an increased efficiency of human cell engraftment, including hematopoeitic stem cells (HSCs) [8, 11]. However, very few human RBCs are generated in the recipient mice following engraftment of human HSCs [12]. Expressing human cytokines interleukin (IL)-3 and erythropoietin (EPO) in NSG recipient mice leads to an improved reconstitution of human RBCs [13]. Using these improved humanized mice, we show here that *P*. *falciparum* can cause multiple cycles of infection without any myelodepletion and without human RBC supplementation.

## Results

### *P*. *falciparum* can infect and multiply in human RBCs generated in mice

Mice with >40% of human leukocyte reconstitution were injected with plasmids encoding human IL-3 and EPO. One month later mice were analysed for human RBC reconstitution. The human RBC reconstitution in mice used in this study ranged between 1.6 and 4.0% (**S1 Table**). Interestingly reticulocytes account for 10 to 30% of human RBCs as measured by staining the blood cells with anti-glycophorin antibodies and thiozol orange (**S1 Fig**). 100 μl of whole blood from these mice were used for *ex vivo* infection with 1x10^6^ mature *P*. *falciparum* schizonts (3D7 strain). Microscopic analysis of blood smears at 16 h showed ring stage infection (**S2A Fig**) with a parasitemia of 0.02 to 1.6% (or 1 to 59.3% when normalised to human RBC frequency in the whole blood). Different batches of mice as well as stem cells from different sources were used for each batch. The following factors may be related to the high variation in parasitemia (**S1 Table**), i.e. genetic background of the donor, the quality of the hematopoietic stem cells, the efficiency of the hydrodynamic injection and therefore the IL-3/EPO expression. The *ex vivo* culture was maintained for multiple cycles and different stages of parasite development, including early ring, late ring, trophozoites, and schizonts, were observed over time (**S2B–S2F Fig**). Viable merozoites were produced as indicated by reinvasion of new RBCs at 64 h after the culture was initiated (**S2G Fig**). In contrast, blood from control NSG mice (recipient mice without any human hematopoietic stem cells engraftment) did not show any infection.

**Fig 1:**
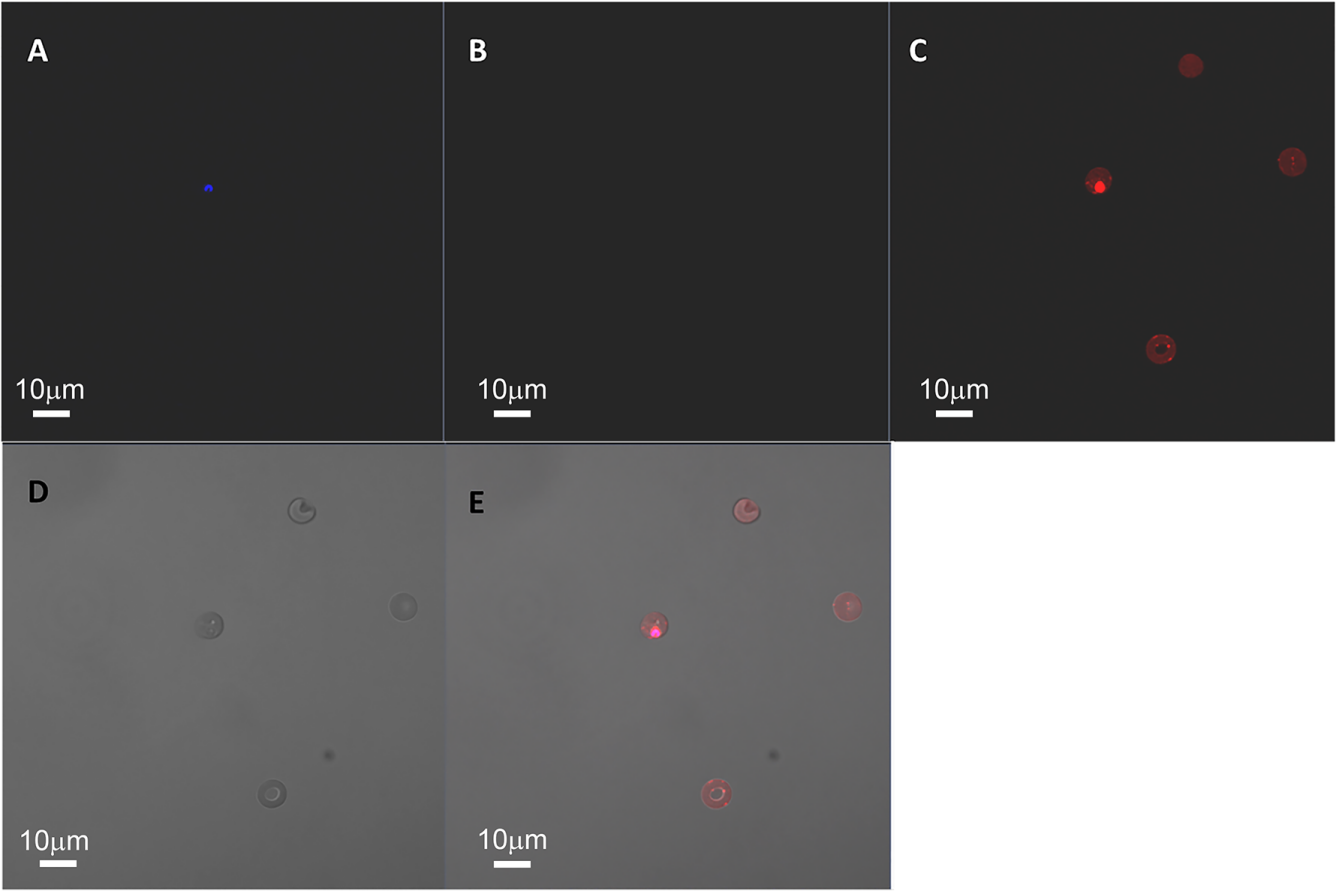
Infection of *P*. *falciparum* (3D7) in *de novo* generated human RBCs. RBCs from humanized mice and purified schizonts were stained with Red CMTPX and Green CMFDA dyes, respectively. After the overnight culture, the parasites were stained with DAPI and imaged under fluorescent microscope (Panel A). Panel B and C represent the images taken under Green (contaminant RBC from culture) and Red (humanized mice RBC) channels. A ring stage parasite can be seen in red stained RBC (Panel C) indicating the infection of *de novo* generated human RBC in humanized mice. Panel D and E represent the bright field and composite images respectively.

In order to confirm that the infected RBCs are in fact from humanized mouse origin and not from contaminant RBCs carried along with purified schizonts, we performed a double staining experiment. RBCs from humanized mice and *in vitro* parasite culture (source of schizonts) were stained with CellTracker Red CMTPX and Green CMFDA (Life Technologies) fluorescent dyes, respectively. These dyes freely pass through cell membranes into the cells, where they transform into a cell-impermeable, fluorescent product which are retained for several days. After overnight culture of the purified schizonts with blood from humanized mice, the parasites were stained with DAPI and imaged under fluorescent microscope. The parasites (blue) were observed in RBCs stained with red confirming that the parasites invaded only the *de novo* generated human RBC from mice (**Fig 1**).

A previous study has shown that the *P*. *falciparum* 7G8 and Camp strains can invade mouse erythrocytes but development was blocked at the early ring stage, leading to the lysis of the infected RBCs [14]. In our study, multiple rounds of infection were observed with 3D7 strain in blood from humanized mice but not from NSG mice, suggesting that the infected cells are human RBCs only. Furthermore, the infected RBCs were stained positive for human glycophorin a/b (**S3 Fig**). These results show that *P*. *falciparum* can infect, develop and mature in the *de novo* generated human RBCs in mice.

**Fig 2:**
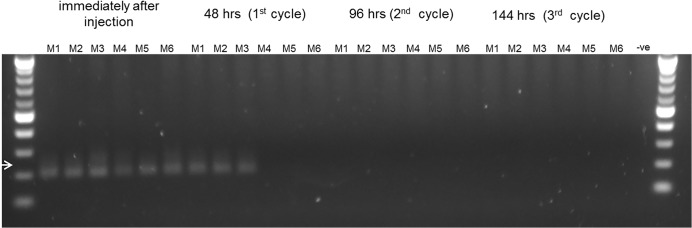
Detection of *P*. *falciparum* (3D7) infection in humanized mice by nested PCR. Humanized mice were injected intravenously with either live ring stage parasites (M1, M2 and M3) or killed ring stage parasites (M4, M5 and M6). 20 μl blood samples were collected immediately after injection or 48, 96 and 144 h after injection and the presence of parasite DNA was assayed by nested PCR. Arrow indicates 205 bp PCR product. Genomic DNA prepared from blood of an uninfected mouse was used as negative (-ve) control.

To test whether *de novo* generated human RBCs can be directly infected *in vivo*, humanized mice were injected with 2x10^7^ purified schizonts (3D7) intravenously. The amount of injected schizonts was calculated based on the *ex vivo* infection using 1x10^6^ schizonts in 100 μl blood from humanized mice and approximately 2 ml blood per mouse. However, no *in vivo* infection was detected in the circulation by microscopy, flow cytometry or nested PCR. The nested PCR amplifies the conserved sequences of the small-subunit rRNA (ssrRNA) gene [15] and would allow us to detect very low levels of circulating parasites. This lack of infection could be due to the fact that *P*. *falciparum* schizonts-infected RBCs are more rigid than the ring stage parasite-infected RBCs [16, 17], and therefore are more likely to be retained in the spleen before they rupture and release the merozoites that can invade new RBCs. Therefore, we injected humanized mice with ring stage parasites obtained from *ex vivo* infection of whole blood from humanized mice. The maximal parasitemia in the *ex vivo* cultures was 1.6% (among total RBCs) and therefore the number of injected ring stage parasites in 100 μl was about 8x10^6^ [0.016 x 5x10^8^ (total RBC in 100 μl mouse blood)]. Parasites were detected by nested PCR at 48 h post infection (**Fig 2**). Because nuclear materials from killed parasites are rapidly cleared from the circulation and do not contribute significantly to PCR amplification [18], the result indicate *P*. *falciparum* infection in humanized mice but at low efficiency.

### Different strains of *P*. *falciparum* show variation in their ability to infect RBCs from humanized mice

**Fig 3:**
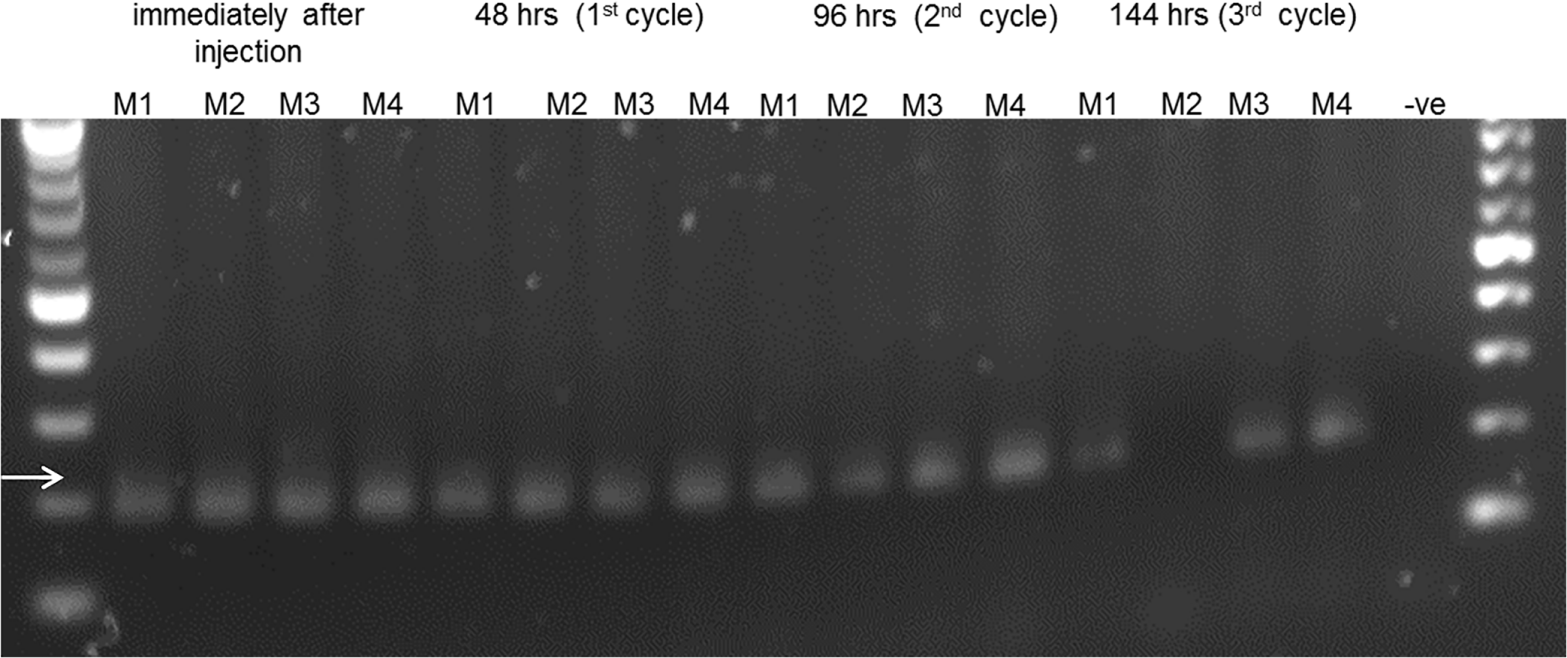
Infection of *ex vivo* cultured *P*. *falciparum* K1 strain in humanized mice. Humanized mice were infected with *ex vivo* cultured *P*. *falciparum* K1 ring stage parasites and the parasite PCR product (indicated by arrow) was determined using nested PCR at the indicated time points after parasite injection. 3 out of 4 mice (M1, M3 and M4) showed infected human RBCs until the 3^rd^ cycle. Genomic DNA prepared from blood of an uninfected mouse was used as negative (-ve) control.

To identify a parasite strain that can infect humanized mice more efficiently, we screened various *P*. *falciparum* strains along with a knob-less clone of 3D7 (3D7KL) and KAHRP k/o parasites [19]. Ring stage parasites were prepared from *ex vivo* culture with 100 μl blood from humanized mice as described above. The level of parasitemia was considered low but detectable when at least 1 parasite could be detected in 100 microscopic fields. Humanized mice were injected intravenously with the culture containing the ring stage parasites and infection was analyzed at 48, 96 and 144 h after injection. Among the 9 strains tested, parasite PCR products for FCR3 and T994 strains were detected in blood samples taken immediately after injection but not after 48 h. Parasite PCR products for DD2, KAHRP k/o, 7G8, and W2mef strains were detected at 48 h after injection. Parasite PCR products for HB3, K1 and 3D7KL strains were detected at 96 h after injection. At 144 h after injection, parasite PCR products were detected in one of the two K1 and HB3 samples (**Fig 3** and **S4 Fig**), suggesting that these parasites can infect humanized mice for three cycles.

### *P*. *falciparum* K1 SMG01 infection in humanized mice can be detected by microscopy for multiple cycles

To further increase efficiency of infection, i.e., detectable by microscopy, K1 strain was selected by *in vivo* adaptation in NSG mice supplemented with human RBCs. Human RBC-supplemented NSG mice infected with 2x10^7^ ring stage parasites showed 2% parasitemia 48 h after injection. Afterwards, however, parasitemia declined to undetectable level. After 16 days, parasitemia was detected again by microscopy and reached 2% by day 20. The details of experimental procedures of *in vivo* adaptation are shown in **S5 Fig**. Using the adapted K1 strain (named SMG01) to infect new human RBC-supplemented NSG mice, parasitemia was consistently detected for over 10 days (**S6 Fig**).

**Fig 4:**
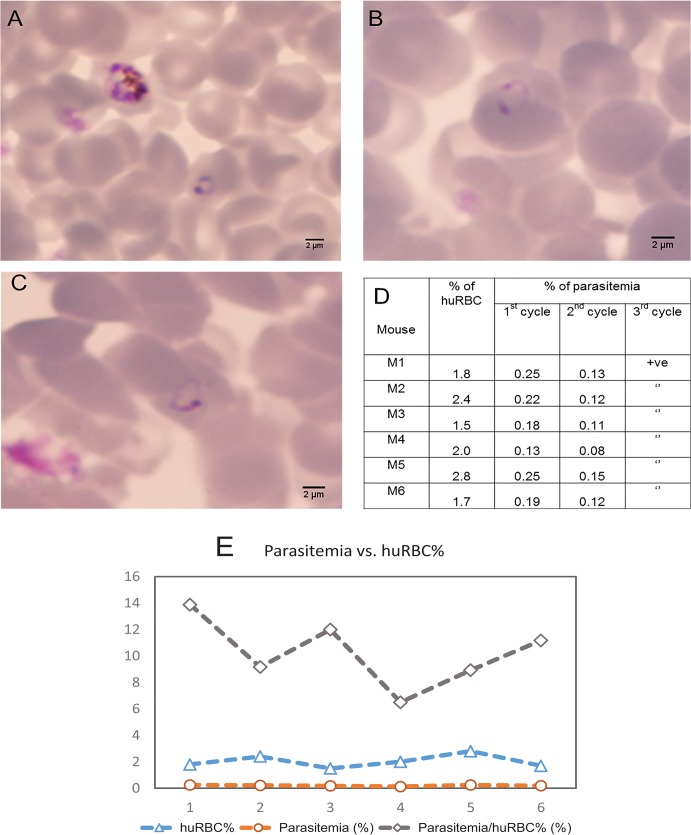
Direct infection of humanized mice by selected *P*. *falciparum* SMG01. Humanized mice were injected intravenously with the ring stage parasites of the *in vivo* adapted K1 *P*. *falciparum* strain SMG01. Thin blood smear was made and stained with Giemsa. Representative ring stage parasites are shown at 48 h (A), 96 h (B) and 144 h (C) after infection. Quantification of parasitemia is presented in (D). Positive (+ve) reading means that the level of parasitemia was low but detectable when at least 1 parasite could be detected in 100 microscopic fields. The parasitemia versus human RBC percentage (huRBC%) is shown in (E), where the average parasitemia/huRBC% is 10.3% ± 2.4%.

We then examined whether SMG01 parasites can generate microscopy-detectable infection in humanized mice where human RBCs are generated *de novo*. Unlike the parental K1 strain, SMG01 ring stage parasites were detected for three cycles by Giemsa staining of blood smear (**Fig 4A–4C**). The parasitemia ranged between 0.13 and 0.25% at the first cycle of infection, fell between 0.08 and 0.15% at the second cycle, and dropped to barely detectable levels at the third cycle of infection (**Fig 4D**). The parasitemia (%) versus human RBC percentage (huRBC%) is shown in **Fig 4E**, where the parasitemia/huRBC% (i.e., the parasitemia with respect to human RBCs) is 10.3% ± 2.4%. The low level of parasitemia could be attributed to the fact that there were only 1.5 to 2.8% human RBCs in the circulation. As a result, short-lived free merozoites are probably cleared before they have a chance to invade another human RBC to sustain the infection. Nevertheless, these results show that human RBCs generated *de novo* in mice can be infected by a selected *P*. *falciparum* K1 SMG01 parasite for at least 3 cycles.

## Discussion

In both human RBC-supplemented NSG mice and the humanized mice presented here, some strains of *P*. *falciparum* are able to induce significant levels of infection. In the *in vivo* adaptation process, initial parasite clearance and reappearance after a period of time is observed. These results are in agreement with a previous report [20] and this may be due to the retention of majority of the infected RBCs in the sinus cord of the spleen [21, 22]. Failure to detect newly invaded parasites after injection of schizonts could also be due to the reason that infected RBCs are retained in the spleen due to their increased rigidity. Studies have shown that the deformability of infected RBCs is a key factor affecting their clearance. Because infected RBCs become more rigid as the development of the parasite progresses [16, 17], they are likely being cleared more rapidly [22]. Screening parasite strains and their further adaptation in mice may select for parasites that maintain higher deformability of the infected RBCs, although rigorous and systematic experiments are needed to confirm this possibility. The *in vivo* adaptation of the parasite we used is similar to Angulo-Barturen et al [20]. However we showed infection in *de novo* generated huRBCs in a humanized mouse model. More recently Arnold et al [23] reported a NSG-IV model of malaria parasite infection, which does not require preadaptation of the parasites. However, these mice still required injection of clodronate liposomes along with human blood.

All existing mouse models of *P*. *falciparum* infection rely on supplementing immunodeficient mice with human RBCs and/or the treatment with clodronate liposomes. In comparison, the humanized mouse model used in this study generates *de novo* human RBCs as well as human immune cells from the same donor. For the first time the humanized mouse model presented here allows successful infection of *P*. *falciparum in vivo* without any human RBC supplementation or treating mice with anti-neutrophil antibodies and clodronate liposomes to suppress clearance of human RBCs. The amount of *de novo* generated huRBCs is nevertheless still fairly low. Further improvement of human RBC reconstitution in our model is necessary in order to sustain *in vivo* parasite infection for more than three cycles and significantly increase parasitemia levels. This model, after further development, has the potential to serve as a fully integrated humanized mouse model for studying host immune responses against *P*. *falciparum* in which both the human immune system as well as the human RBCs have the same origin.

## Methods

### Humanized Mice

NSG mice were purchased from the Jackson Laboratories (Bar Harbor, Maine) and maintained under specific pathogen-free conditions in the animal facilities at National University of Singapore, Singapore. Reconstitution of human blood lineage cells was carried out as described previously [13]. Briefly, newborn NSG pups (less than 48 h old) were irradiated with 100 cGy using a Gamma radiation source and injected intracardially with CD34^+^ HSCs from cord blood (1x10^5^ cells/recipient). Mice were analyzed for human leukocyte reconstitution at 10–12 weeks of age. Mice with 40% or more human leukocyte reconstitution in the peripheral blood mononuclear cells (PBMCs) were injected hydrodynamically with plasmids encoding human IL-3 and EPO to improve human RBC reconstitution, as specified in detail in [13]. The level of human RBCs in humanized mice was quantified by staining blood cells with FITC-conjugated anti-human glycophorin a/b antibody (BioLegend, USA). Representative FACS data for human RBC characterization is presented in **S1 Fig**. Mice with a minimum of 1.5% human RBC reconstitution were then used for subsequent experimentation. All studies involving human HSC from cord blood and mice were approved by the institutional review board (IRB) and institutional animal care and use committee (IACUC) of the National University of Singapore. Studies involving mice were also approved by the committee on animal care (CAC) of the Massachusetts Institute of Technology.

### *P*. *falciparum* culture

*P*. *falciparum* strains 3D7, DD2, HB3, K1, KAHRP k/o, 7G8, FCR3, T994 and W2Mef were obtained from Malaria Research and Reference Reagent Resource (MR4) and maintained in leukocyte-free human erythrocytes as described by Trager and Jensen [24]. *P*. *falciparum* 3D7KL (knob-less) clone was generated by limiting dilution and selecting for knob-less clone. This clone does not produce knobs on the infected erythrocyte membrane and has increased deformability compared to the parental line (Li Ang, personal communication). The parasites were treated with trypsin/chymotrypsin or neuraminidase depending on the strain for one h at 37°C with shaking before purification of schizonts in order to prevent the reinvasion of residual RBCs [25]. The late stage schizonts were purified by percoll gradient centrifugation according to Fernandez [26].

### *Ex vivo* infection

100 μl whole blood was collected via facial vein from humanized mice and washed twice with RPMI medium. RBCs were resuspended in 1 ml PRMI medium containing 0.5% heat-inactivated human serum. After addition of 1x10^6^ purified schizonts the mixture was incubated at 37° C for 16 to 64 hrs and then analyzed for infection by Giemsa staining of blood smear.

### Fluorescent staining of RBCs

Whole blood collected from humanized mice via facial vein bleeding was washed with RPMI and incubated with 10 μM CellTracker Red CMTPX dye (Life Technologies) for 30 minutes at 37°C under shaking condition. Similarly, the *in vitro P*. *falciparum* culture was also stained with CellTracker Green CMFDA dye. The humanized mouse RBCs and the *P*. *falciparum* culture were washed with RPMI and the schizonts were purified. After co-culture of schizonts with blood from humanized mice overnight, the parasites were stained with DAPI and imaged under fluorescent microscope.

### RBC supplementation and adaptation of parasites

For RBC supplementation, human blood was pelleted and resuspended in equal volume of RPMI medium containing 50% heat-inactivated human serum (Invitrogen). 4 to 6 weeks old male or female NSG mice were each injected daily with 1 ml human RBCs intraperitoneally. When human RBCs reached ~20% as determined by anti-glycophorin a/b staining (after about 7–10 days), mice were used for *in vivo* adaptation of parasites as described by Angulo-Barturen et al [20]. To infect RBC-supplemented NSG mice, *P*. *falciparum* K1 strain of the parasite was cultured with human RBCs as described [24]. At the peak of ring stage infection, the culture was harvested and 2x10^7^ ring stage parasites were injected intravenously per recipient mice.

### Infection of humanized mice

Parasite infection of humanized mice was carried out in three ways. In the first approach, purified schizonts were injected into humanized mice intravenously (2x10^7^ schizonts per mouse). In the second approach, 100 μl whole blood from humanized mice containing human RBCs was infected with 1x10^6^ schizonts overnight. The parasitemia in these infected cultures ranged between 0.02 and 1.6% (correspond to 2x10^4^ to 8x10^6^ parasites in total). The cultures were washed with incomplete RPMI media and then injected intravenously into the same mouse from which the blood was collected. In the third approach, the selected parasite K1 strain was used to infect human RBC-supplemented NSG mice as above. When parasitemia reached ~2%, the blood was harvested, treated with tryspin/chymotrypsin and 2x10^7^ ring stage parasites were injected intravenously into humanized mice directly or from a frozen stock of K1 adapted parasite strain.

### Parasite detection by nested PCR and immunofluorescence assay (IFA)

Parasite genomic DNA was extracted using the Easy-DNA Kit (Invitrogen) following the manufacturer’s protocol. Nested PCR was performed as described by Snounou et al [15]. Genomic DNA prepared from blood of uninfected mice was used as negative control in all PCR reactions. IFA was carried out as described by Blackman [27].

## Supporting Information

S1 Fig Reticulocytes among total human RBCs in humanized mice. The top panel shows representative percentage of human RBCs in 4 different mice. The bottom panel shows the reticulocytes among human RBCs in the same mice. RBCs are enucleated and the RNA slowly degrades as the cells mature. However reticulocytes (young RBCs) carry residual RNAs which can be stained with Thiozol Orange (TO). Since leukocytes are removed from the samples only reticulocytes will be stained. To differentiate human reticulocytes they are co-stained with anti glycophorin a/b (GPA/B) antibodies.
(PDF)

S2 Fig *Ex vivo* infection of human RBCs from humanized mice by *P*. *falciparum* (3D7). Parasites and whole blood from humanized mice were mixed and co-cultured. A thin smear of the culture was stained with Giemsa at different time points and visualized for infected RBCs. A) Representative image of Giemsa staining at 16 h after starting the culture showing two infected RBCs with ring stage parasites. B-G) Giemsa stained parasites showing different developmental stages: purified schizonts from *in vitro* culture (B), newly invaded early ring stage at 16 h (C), late ring at 24 h (D), trophozoite stage at 32 h (E), schizont stage at 48 h (F), and ring stage parasite 64 h after reinvasion of new uninfected RBCs (G). Representative data from 6 mice are shown.
(PDF)

S3 Fig *P*. *falciparum* infects only human RBCs from humanized mice blood. Whole blood from humanized mice were cultured with 3D7 parasites and thin smear of the culture was stained with anti-human glycophorin a/b antibody and DAPI to stain the parasites. Shown are representative images of DAPI (parasite) stain (A), anti-human glycophorin a/b stain (B), and merged image (C). (D) is overexposure of (B) to visualize both human and mouse RBCs.
(PDF)

S4 Fig Infection of humanized mice with different *P*. *falciparum* strains. *P*. *falciparum* strains DD2, HB3, K1 (A), KAHRP k/o, 7G8, FCR3 (B), T994, W2Mef and 3D7KL (C) were used to infect humanized mice and the parasite PCR products with 205 bp size (indicated by arrow) were detected by nested PCR at the indicated time points after parasite infection. Parasite PCR products were detected in one of the two K1 and HB3 samples at 3rd infection cycle whereas in other strains infected RBCs can only be detected until the 1^st^ cycle of blood stage infection. The last lane represents negative (-ve) control for which genomic DNA prepared from blood was used as template.
(PDF)

S5 Fig *In vivo* adaptation of *P*. *falciparum* K1 strain in NSG mice supplemented with human RBCs. NSG mice (M1 and M2) are supplemented daily with human RBCs by intraperitoneal injection. Antibodies to glycophorin a/b (conjugated to FITC) was used to stain the human RBCs for quantification. When human RBC reconstitution reaches about 20% (on day 9 as shown above), NSG mice were infected with 2x10^7^ ring stage parasites of *P*. *falciparum* K1 strain. The supplementation of human RBCs was continued throughout the experiment and an increase in human RBC reconstitution up to 89% could be seen in M1 on day 28. 48h after infection with the parasite, a parasitemia level of 2% was detected. Later on parasitemia, however, decreased to undetectable levels. 16 days after infection parasitemia became detectable by microscopy again and reached 2% by day 20. This adapted *P*. *falciparum* K1 strain named SMG01 was used for further studies.
(PDF)

S6 Fig Infection of human RBC supplemented NSG mice with adapted *P*. *falciparum* K1 parasite strain SMG01. The selected and *in vivo* adapted parasite strain SMG01 can be detected consistently in human RBC-supplemented NSG mice.
(PDF)

S1 Table *Ex vivo* infection of human RBCs from humanized mice by *P*. *falciparum* (3D7). Total human RBCs and parasitemia in 14 mice are shown. Parasitemia ranged between 0.02% (mouse 4) and 1.6% (mouse 6) or 1% to 59.3% when normalized to human RBCs.
(PDF)
