# Supplementary material for: De Novo Generated Human Red Blood Cells in Humanized Mice Support Plasmodium falciparum Infection
Source: PLoS One. 2015 Jun 22;10(6):e0129825. doi: 10.1371/journal.pone.0129825 (PMC4476714; doi:10.1371/journal.pone.0129825)
Supplement: S1 Fig — The top panel shows representative percentage of human RBCs in 4 different mice. The bottom panel shows the reticulocytes among human RBCs in the same mice. RBCs are enucleated and the RNA slowly degrades as the cells mature. However reticulocytes (young RBCs) carry residual RNAs which can be stained with Thiozol Orange (TO). Since leukocytes are removed from the samples only reticulocytes will be stained. To differentiate human reticulocytes they are co-stained with anti glycophorin a/b (GPA/B) antibodies. (PDF) [file pone.0129825.s001.pdf]

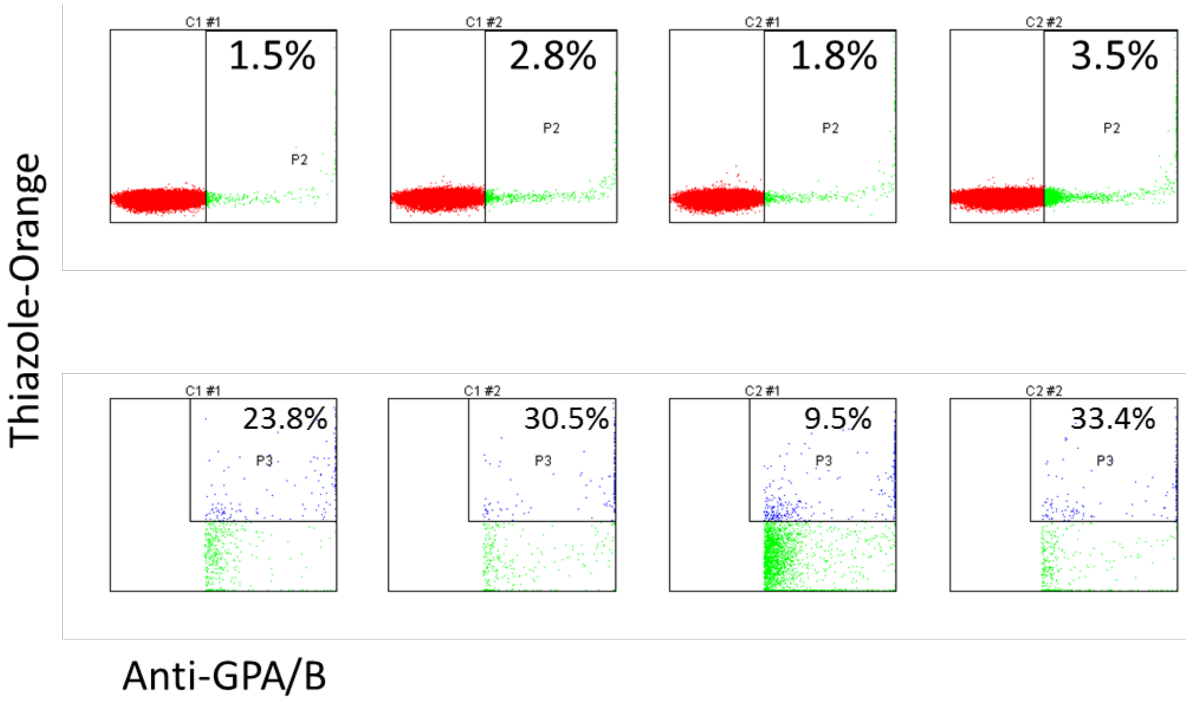


**S1 Fig. Reticulocytes among total human RBCs in humanized mice.** The top panel shows representative percentage of human RBCs in 4 different mice. The bottom panel shows the reticulocytes among human RBCs in the same mice. RBCs are enucleated and the RNA slowly degrades as the cells mature. However reticulocytes (young RBCs) carry residual RNAs which can be stained with Thiozol Orange (TO). Since leukocytes are removed from the samples only reticulocytes will be stained. To differentiate human reticulocytes they are co-stained with anti glycophorin a/b (GPA/B) antibodies.
