# Supplementary material for: De Novo Generated Human Red Blood Cells in Humanized Mice Support Plasmodium falciparum Infection
Source: PLoS One. 2015 Jun 22;10(6):e0129825. doi: 10.1371/journal.pone.0129825 (PMC4476714; doi:10.1371/journal.pone.0129825)
Supplement: S2 Fig — Parasites and whole blood from humanized mice were mixed and co-cultured. A thin smear of the culture was stained with Giemsa at different time points and visualized for infected RBCs. A) Representative image of Giemsa staining at 16 h after starting the culture showing two infected RBCs with ring stage parasites. B-G) Giemsa stained parasites showing different developmental stages: purified schizonts from in vitro culture (B), newly invaded early ring stage at 16 h (C), late ring at 24 h (D), trophozoite stage at 32 h (E), schizont stage at 48 h (F), and ring stage parasite 64 h after reinvasion of new uninfected RBCs (G). Representative data from 6 mice are shown. (PDF) [file pone.0129825.s002.pdf]

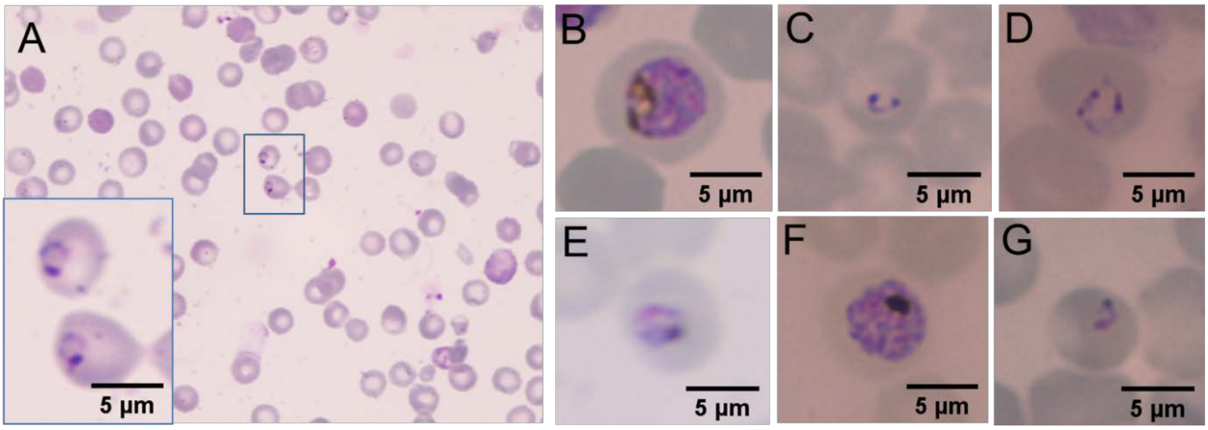

S2 Fig. *Ex vivo* infection of human RBCs from humanized mice by *P. falciparum* (3D7). Parasites and whole blood from humanized mice were mixed and co-cultured. A thin smear of the culture was stained with Giemsa at different time points and visualized for infected RBCs. A) Representative image of Giemsa staining at 16 h after starting the culture showing two infected RBCs with ring stage parasites. B-G) Giemsa stained parasites showing different developmental stages: purified schizonts from *in vitro* culture (B), newly invaded early ring stage at 16 h (C), late ring at 24 h (D), trophozoite stage at 32 h (E), schizont stage at 48 h (F), and ring stage parasite 64 h after reinvasion of new uninfected RBCs (G). Representative data from 6 mice are shown.
