# Supplementary material for: De Novo Generated Human Red Blood Cells in Humanized Mice Support Plasmodium falciparum Infection
Source: PLoS One. 2015 Jun 22;10(6):e0129825. doi: 10.1371/journal.pone.0129825 (PMC4476714; doi:10.1371/journal.pone.0129825)
Supplement: S3 Fig — Whole blood from humanized mice were cultured with 3D7 parasites and thin smear of the culture was stained with anti-human glycophorin a/b antibody and DAPI to stain the parasites. Shown are representative images of DAPI (parasite) stain (A), anti-human glycophorin a/b stain (B), and merged image (C). (D) is overexposure of (B) to visualize both human and mouse RBCs. (PDF) [file pone.0129825.s003.pdf]

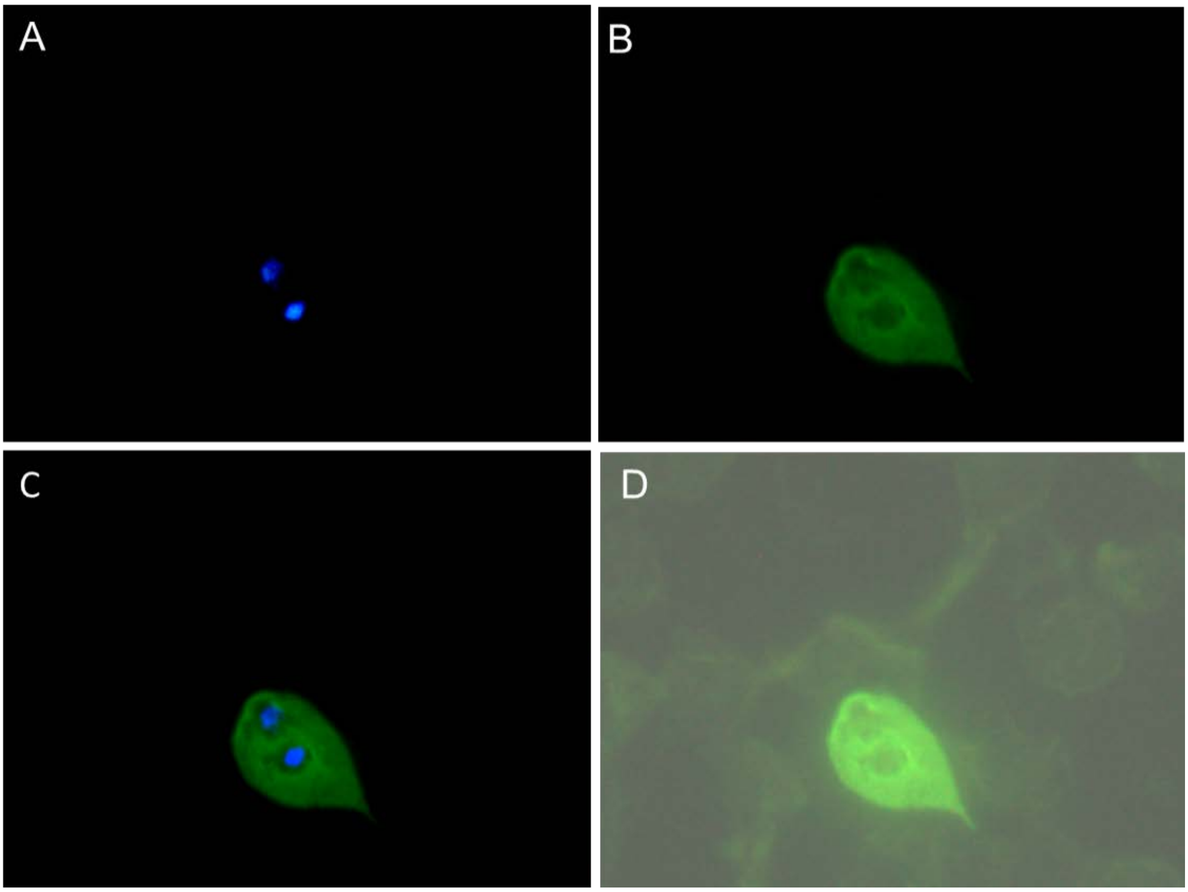

S3 Fig. *P. falciparum* infects only human RBCs from humanized mice blood. Whole blood from humanized mice were cultured with 3D7 parasites and thin smear of the culture was stained with anti-human glycophorin a/b antibody and DAPI to stain the parasites. Shown are representative images of DAPI (parasite) stain (A), anti-human glycophorin a/b stain (B), and merged image (C). (D) is overexposure of (B) to visualize both human and mouse RBCs.
