# Supplementary material for: De Novo Generated Human Red Blood Cells in Humanized Mice Support Plasmodium falciparum Infection
Source: PLoS One. 2015 Jun 22;10(6):e0129825. doi: 10.1371/journal.pone.0129825 (PMC4476714; doi:10.1371/journal.pone.0129825)
Supplement: S4 Fig — P. falciparum strains DD2, HB3, K1 (A), KAHRP k/o, 7G8, FCR3 (B), T994, W2Mef and 3D7KL (C) were used to infect humanized mice and the parasite PCR products with 205 bp size (indicated by arrow) were detected by nested PCR at the indicated time points after parasite infection. Parasite PCR products were detected in one of the two K1 and HB3 samples at 3rd infection cycle whereas in other strains infected RBCs can only be detected until the 1st cycle of blood stage infection. The last lane represents negative (-ve) control for which genomic DNA prepared from blood was used as template. (PDF) [file pone.0129825.s004.pdf]

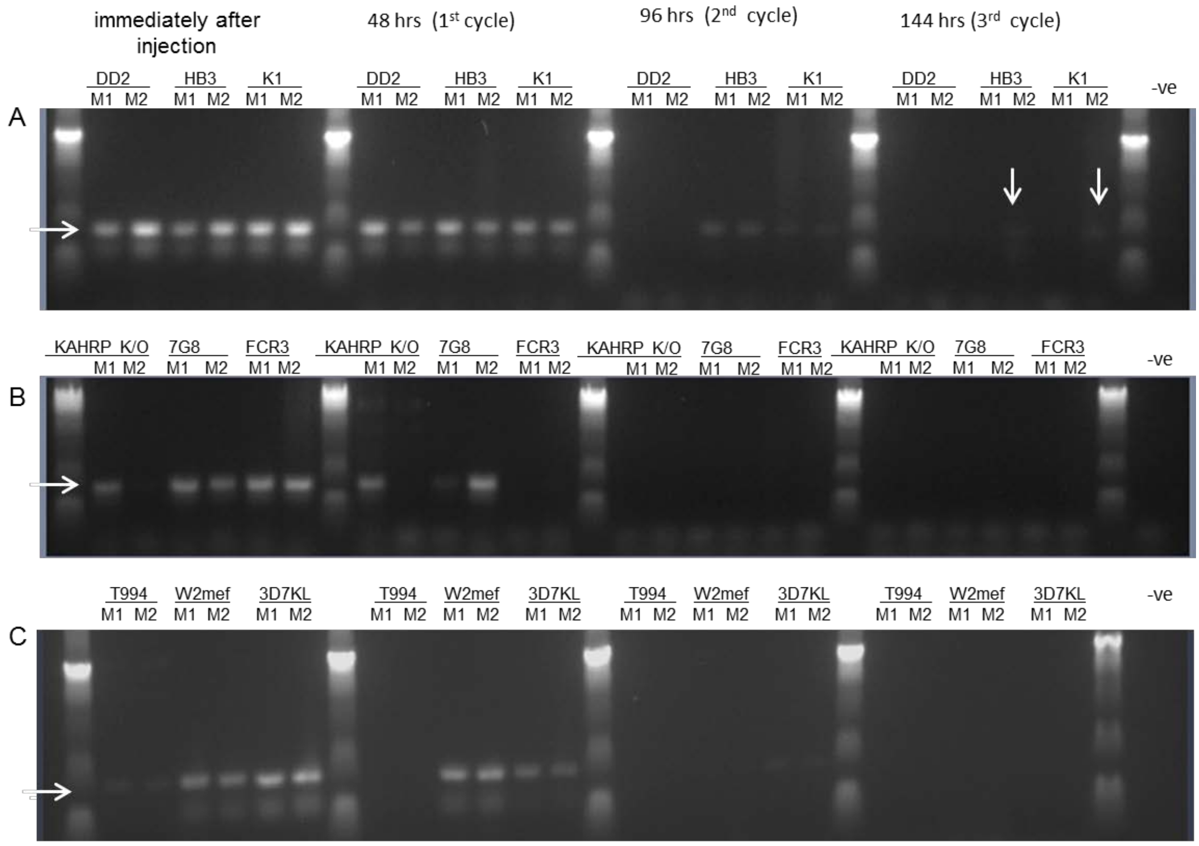

S4 Fig. **Infection of humanized mice with different *P. falciparum* strains.** *P. falciparum* strains DD2, HB3, K1 (A), KAHRP k/o, 7G8, FCR3 (B), T994, W2Mef and 3D7KL (C) were used to infect humanized mice and the parasite PCR products with 205 bp size (indicated by arrow) were detected by nested PCR at the indicated time points after parasite infection. Parasite PCR products were detected in one of the two K1 and HB3 samples at 3rd infection cycle whereas in other strains infected RBCs can only be detected until the 1st cycle of blood stage infection. The last lane represents negative (-ve) control for which genomic DNA prepared from blood was used as template.
