# Supplementary material for: De Novo Generated Human Red Blood Cells in Humanized Mice Support Plasmodium falciparum Infection
Source: PLoS One. 2015 Jun 22;10(6):e0129825. doi: 10.1371/journal.pone.0129825 (PMC4476714; doi:10.1371/journal.pone.0129825)
Supplement: S5 Fig — NSG mice (M1 and M2) are supplemented daily with human RBCs by intraperitoneal injection. Antibodies to glycophorin a/b (conjugated to FITC) was used to stain the human RBCs for quantification. When human RBC reconstitution reaches about 20% (on day 9 as shown above), NSG mice were infected with 2x10^7^ ring stage parasites of P. falciparum K1 strain. The supplementation of human RBCs was continued throughout the experiment and an increase in human RBC reconstitution up to 89% could be seen in M1 on day 28. 48h after infection with the parasite, a parasitemia level of 2% was detected. Later on parasitemia, however, decreased to undetectable levels. 16 days after infection parasitemia became detectable by microscopy again and reached 2% by day 20. This adapted P. falciparum K1 strain named SMG01 was used for further studies. (PDF) [file pone.0129825.s005.pdf]

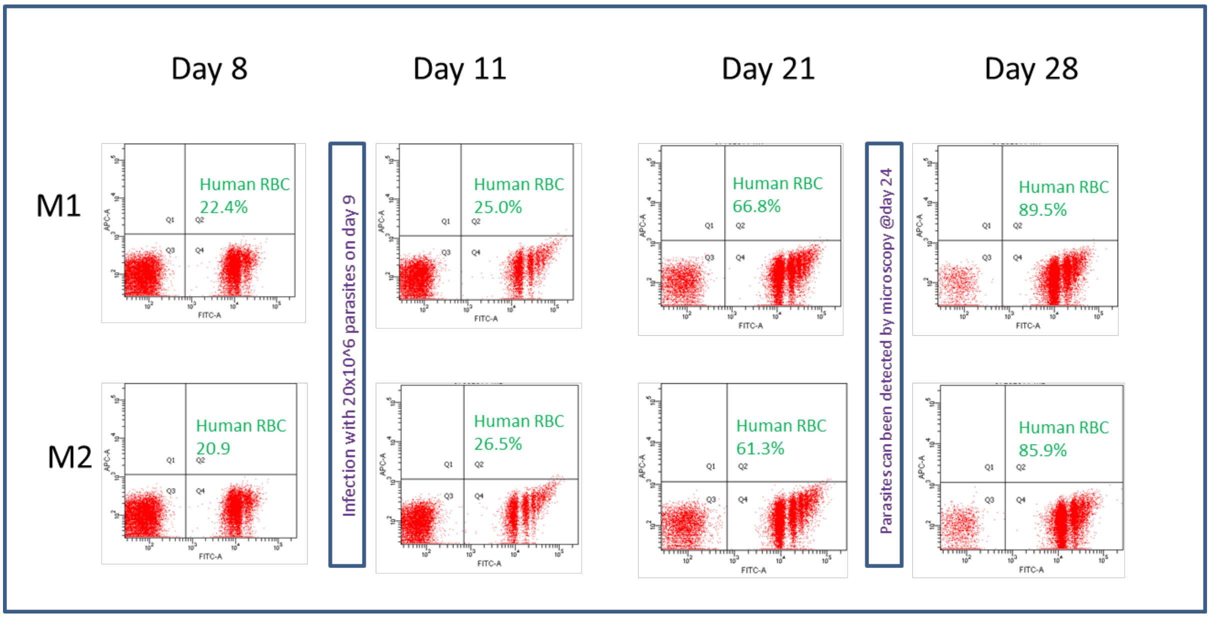

S5 Fig. *In vivo* adaptation of *P. falciparum* K1 strain in NSG mice supplemented with human RBCs. NSG mice (M1 and M2) are supplemented daily with human RBCs by intraperitoneal injection. Antibodies to glycophorin a/b (conjugated to FITC) was used to stain the human RBCs for quantification. When human RBC reconstitution reaches about 20% (on day 9 as shown above), NSG mice were infected with $2x10^7$ ring stage parasites of *P. falciparum* K1 strain. The supplementation of human RBCs was continued throughout the experiment and an increase in human RBC reconstitution up to 89% could be seen in M1 on day 28. 48h after infection with the parasite, a parasitemia level of 2% was detected. Later on parasitemia, however, decreased to undetectable levels. 16 days after infection parasitemia became detectable by microscopy again and reached 2% by day 20. This adapted *P. falciparum* K1 strain named SMG01 was used for further studies.
