# Supplementary material for: De Novo Generated Human Red Blood Cells in Humanized Mice Support Plasmodium falciparum Infection
Source: PLoS One. 2015 Jun 22;10(6):e0129825. doi: 10.1371/journal.pone.0129825 (PMC4476714; doi:10.1371/journal.pone.0129825)
Supplement: S6 Fig — The selected and in vivo adapted parasite strain SMG01 can be detected consistently in human RBC-supplemented NSG mice. (PDF) [file pone.0129825.s006.pdf]

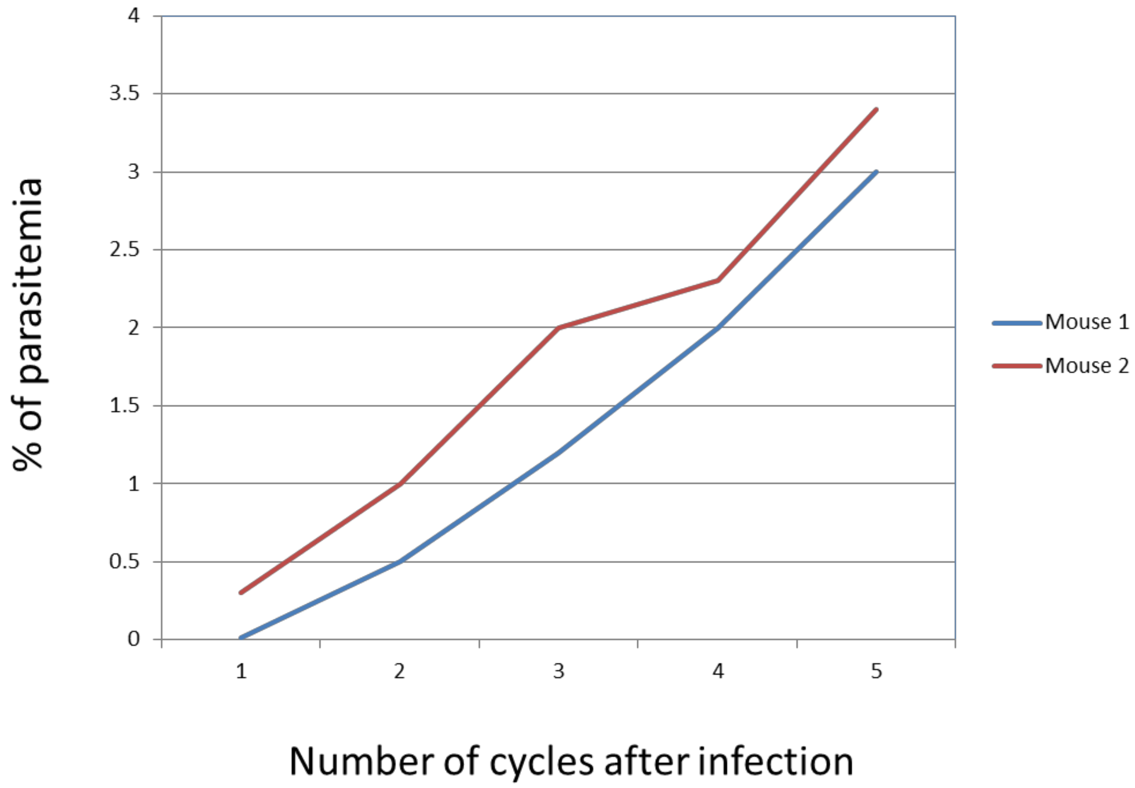


**S6 Fig. Infection of human RBC supplemented NSG mice with adapted *P. falciparum* K1 parasite strain SMG01**. The selected and *in vivo* adapted parasite strain SMG01 can be detected consistently in human RBC-supplemented NSG mice.
