# Supplementary material for: De Novo Generated Human Red Blood Cells in Humanized Mice Support Plasmodium falciparum Infection
Source: PLoS One. 2015 Jun 22;10(6):e0129825. doi: 10.1371/journal.pone.0129825 (PMC4476714; doi:10.1371/journal.pone.0129825)
Supplement: S1 Table — Total human RBCs and parasitemia in 14 mice are shown. Parasitemia ranged between 0.02% (mouse 4) and 1.6% (mouse 6) or 1% to 59.3% when normalized to human RBCs. (PDF) [file pone.0129825.s007.pdf]

Mouse	% of huRBC	% of parasitemia	
		Total blood	Normalized to human RBC
M1	3	0.3	10
M2	3	0.2	6.6
M3	3	0.2	6.6
M4	2	0.02	1
M5	4	0.58	14.5
M6	3.5	1.6	45.7
M7	3	0.3	10
M8	3	0.3	10
M9	3	0.2	6.6
M10	3.7	0.37	10
M11	1.8	0.26	14
M12	1.6	0.95	59.3
M13	1.6	0.76	47.5
M14	2.2	0.6	27

S1 Table. *Ex vivo* infection of human RBCs from humanized mice by *P. falciparum* (3D7). Total human RBCs and parasitemia in 14 mice are shown. Parasitemia ranged between 0.02% (mouse 4) and 1.6% (mouse 6) or 1% to 59.3% when normalized to human RBCs.
